# Vanadium Dioxide Circuits Emulate Neurological Disorders

**DOI:** 10.3389/fnins.2018.00856

**Published:** 2018-11-30

**Authors:** Jianqiang Lin, Supratik Guha, Shriram Ramanathan

**Affiliations:** ^1^Center for Nanoscale Materials, Argonne National Laboratory, Lemont, IL, United States; ^2^Institute for Molecular Engineering, University of Chicago, Chicago, IL, United States; ^3^School of Materials Engineering, Purdue University, West Lafayette, IN, United States; ^4^School of Electrical and Computer Engineering, Purdue University, West Lafayette, IN, United States

**Keywords:** strongly correlated systems, VO_2_, central nervous system diseases, Hodgkin-Huxley model, artificial neurons

## Abstract

Information in the central nervous system (CNS) is conducted via electrical signals known as action potentials and is encoded in time. Several neurological disorders including depression, Attention Deficit Hyperactivity Disorder (ADHD), originate in faulty brain signaling frequencies. Here, we present a Hodgkin-Huxley model analog for a strongly correlated VO_2_ artificial neuron system that undergoes an electrically-driven insulator-metal transition. We demonstrate that tuning of the insulating phase resistance in VO_2_ threshold switch circuits can enable direct mimicry of neuronal origins of disorders in the CNS. The results introduce use of circuits based on quantum materials as complementary to model animal studies for neuroscience, especially when precise measurements of local electrical properties or competing parallel paths for conduction in complex neural circuits can be a challenge to identify onset of breakdown or diagnose early symptoms of disease.

## Introduction

Action potentials (AP) are generated in neurons and propagated to other neurons via synapses (Hodgkin and Huxley, 1952; Kandel, 2012). The frequency of the spikes carries information and is critical for brain function. How frequently neurons spike for a given stimulus and whether or not they are able to travel without losing signal strength dictate normal vs. abnormal brain function (Salinas and Sejnowski, 2001; Bartzokis, 2005; Wulff et al., 2009). Alteration in neural oscillations caused by abnormal excitation of action potential has been found to play an important role in a number of neurological disorders. Many research works on molecular neurophysiology have suggested the correlation of pathologically altered action potential excitability. Various neurological diseases are briefly summarized as follows.

Wu. et al. reported that the dysfunctional calcium channel in mutant mouse model is associated with the hypokalemic periodic paralysis which is a form of paroxysmal weakness that occurs in motor neuron disease (Wu, 2012). Research has shown that the Alzheimer's disease can occur due to disruption of neuronal excitability. As examples, Chakroborty et al. showed that increase of frequency and amplitude of AP due to certain protein channel dysregulation results in excitability impairment, with which the Triple Tg expression model is developed (Chakroborty et al., 2009; Santos et al., 2010). Drug addiction is also strongly related to abnormal excitation of action potential. Kourrich et al. revealed the relationship between drug addiction and brain activity. They found that neurons subjected to certain dose of Cocaine will fire about 30% faster at high input current, and 200% faster at low input current (Kourrich et al., 2015). *Global Burden of Disease Study* revealed that major depression was the second largest cause of disability (estimated by the loss of productivity from the disease) and it affected approximately 300 million people worldwide in 2010 (Vos et al., 2012). Friedman et al. showed that the midbrain dopamine neurons have played important in certain depressions. When the dopamine neuron (in mice) fire rate increases by 50% (from 1.6 to 2.4 Hz), the social interaction (measured by a special experiment, see reference) dropped by about 60% (Friedman et al., 2014). Bipolar disorder, also known as manic depression, has been studied in monkeys and it is found that the mental disorder is related to the prefrontal cortical neurons firing and signaling at the molecular level (Birnbaum et al., 2004). Another example in the context of understanding and curing neurological syndromes is the research on neuropathic pain. Researchers sought for treatment for pain by treatments to tune the neuron oscillation frequency (Campbell and Meyer, 2006). These studies and references contained in them strongly indicate the crucial role of controlling the spiking frequencies and action potential generation in order-disorder transitions in biological neural circuits. Besides these examples, other neurological disorders that are resulted from pathologically-altered brain signaling frequencies also include neuromuscular diseases (Younger, 1999; Hutchison et al., 2004; Nelson and Valakh, 2015), ADHD (Brennan and Arnsten, 2008) and etc.

Understanding their origins and the mechanisms to minimize damage to neural pathways is a principal area of study in neuroscience. However, diagnosis of neurological disorder at the molecular level is challenging (Brown et al., 2004). One widely adopted method for neurophysiological measurements is the multiple-electrode recording of the electrical signal of AP spikes in brain tissue (Brown et al., 2004). To-date, neural recording experiments usually involve invasive probing (Kinney et al., 2015) and the *in vivo* measurements are mostly carried out on small animals such as mice (Schulz et al., 2014; Barry, 2015). Artificial circuits that mimic desired signal propagation characteristics along neurons and can provide parametric information on normal-abnormal signaling transitions from electrical properties of circuit components could be valuable in evaluating or directing animal studies. Here, we propose understanding electrical behavior of neurons and neurological disorders via synthetic circuits comprised of a strongly correlated oxide VO_2_ that undergoes an electrically-driven *insulator-metal transition* (*IMT*).

Oxides have been studied for electronic devices such as resonant tunneling diodes, single-electron transistors, and steep slope switches (Mannhart and Schlom, 2010; Vitale et al., 2015). Among these emerging oxide-based electronic device concepts, phase changing artificial neurons has primarily focused on applications in neuromorphic computing to mimic the leaky-integrate-fire function (Pickett et al., 2013; Lin et al., 2016; Mehonic and Kenyon, 2016; Tuma et al., 2016; Dutta et al., 2017). Here, we present a *Hodgkin-Huxley (HH) model analog* for the intrinsic properties of a solid-state material, VO_2_. The strongly correlated VO_2_ artificial neuron system can undergo an electrically driven IMT akin to the excitable membrane in the biological neuron. Changes in composition of the material synergistically modifies the ground state resistivity, IMT strength defined as resistance ratio in the two phases as well as the threshold voltage required for initiating a phase change. Such material property is designed to capture the Intrinsic Membrane Excitability (IME) in biological neurons, which refers to a neuron's propensity for generating action potential at a given input. Building on this fundamental concept, we demonstrate neuronal function mimicking a vast range of neuron types found in animal brains and simulate an archetypal monosynaptic circuit (e.g., the *knee-jerk reaction*). Long term, our results may help in creating artificial systems to generate knowledge about thresholds for onset for brain disorders due to neuronal malfunction.

## Materials and methods

VO_2_ thin films of 200 nm thickness were deposited on SiO_2_/Si by reactive sputtering at 775 K. The stoichiometry and IMT transition strength in VO_2_ is controlled by the oxygen partial pressure in the sputtering chamber. The IMT occurs at a critical temperature T_c_. IMT transition strength (R_ins_/R_met_) is defined by the ratio of high resistance state (R_ins_, measured at room temperature) and low resistance state (R_met_, measured at above critical transition temperature). In VO_2_, T_c_ is 67°C. The low resistance state is taken at 120°C that is significantly higher than T_c_. Our film growth experiments have shown controllable thermal IMT strength variation from R_ins_/R_met_ >10^5^ to R_ins_/R_met_ = 1 (complete loss of IMT characteristic) (Ha et al., 2013; Zhou and Ramanathan, 2015; Lin et al., 2016). R_ins_ and R_met_ are respectively, the resistivity for the insulating state and metallic state, and is characterized by temperature-dependent Hall measurement. The IMT strength can be controlled by substrate temperature during film deposition, oxygen partial pressure during growth, and the choice of substrates (Savo et al., 2015). In this study, we used a VO_2_ thin film with IMT strength of 2 × 10^5^ (Lin et al., 2017).

**Figure 1:**
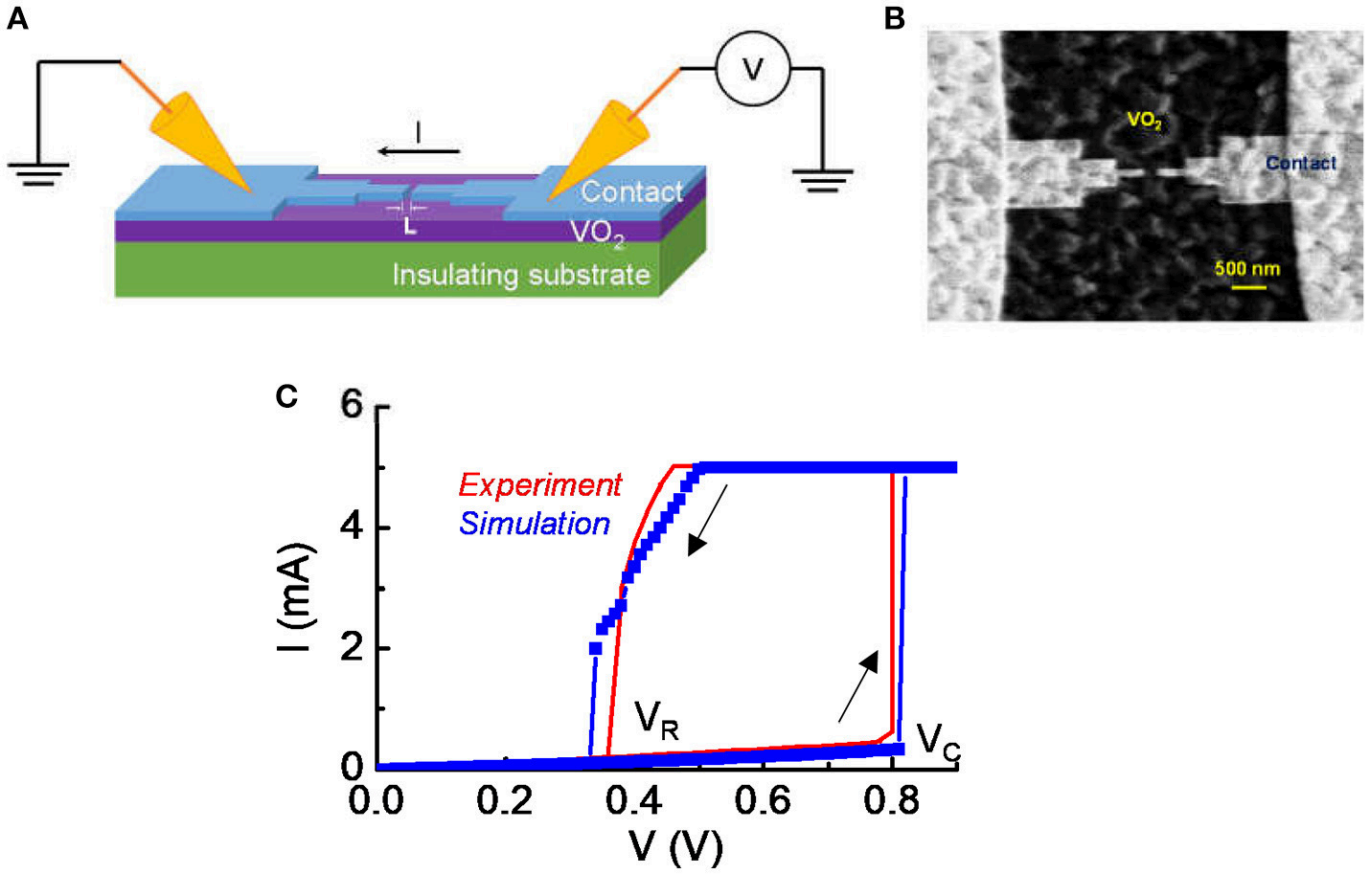
Experimental details for the VO_2_ device. **(A)** The schematic of the VO_2_ devices under DC probing. **(B)** The top view of the lateral VO_2_ device with a “neck-down” layout. The “neck-down” layout leads to lower forward critical transition voltage (V_c_) and lower power in the switching operation. **(C)** The typical current vs. voltage characteristic in DC measurement. The voltage sweep is in the sequence of forward (0–0.9 V) and reverse (0.8–0 V) direction. The switch is reversible. The forward critical voltage and reverse voltage are denoted. A current compliance is set at 5 mA. Simulation shows a nice match with experiment.

After VO_2_ thin film growth, we fabricated lateral device for artificial neuron circuit testing. Electron-beam lithography (EBL) was used to define the length of the VO_2_ device, L. As shown in Figure 1A, L is along the channel direction. Devices of L = 200 nm were used for this study. Ti/Au of thickness 5/100 nm were evaporated and lifted off to form electrical contacts to the VO_2_. A “neck-down” design for the contact were used as illustrated in Figure 1B. The neck-down device drives only a small volume of the VO_2_ into transition and reduces the voltage required to trigger the transition (Lin et al., 2017). In our experiment, the variation in material property is limited. In addition, the degradation of VO_2_ under the experimental condition is largely un-controllable. Therefore, we use a model that has been calibrated with experiment with a circuit simulation approach to derive a systematic understanding for the impact of VO_2_ resistive state on neuron behavior. The model is discussed in the following sessions.

All experiments were carried out at room temperature. The DC sweeping and current-clamp are both performed using Keysight B1500A. Waveforms for the current-clamp experiment were acquired by Keysight Digital Oscilloscope DSO9104A. For DC sweeping, a current compliance is set to 5 mA. For current-clamp response, the IMT device is protected by a series resistance through a circuit board so that the measurement can be repeated reliably by avoiding excess heating and burnout.

Figure 1A shows the schematic of the VO_2_ devices under DC probing and Figure 1B is the top view of the lateral VO_2_ device with a “neck-down” layout. The “neck-down” layout is used to minimize the volume of VO_2_ that undergoes transition (Lin et al., 2017). It leads to lower critical transition voltage (V_c_) and lower power in the switching operation. The spacing between two contacts is L = 200 nm for the device being studied in this work. Figure 1C shows a measured current vs. voltage characteristic under DC condition. A hysteresis sweep is performed and the switch between insulator-state and metal-state is reversible if the operation satisfies the safe criteria introduced in Lin et al. (2017).

**Figure 2:**
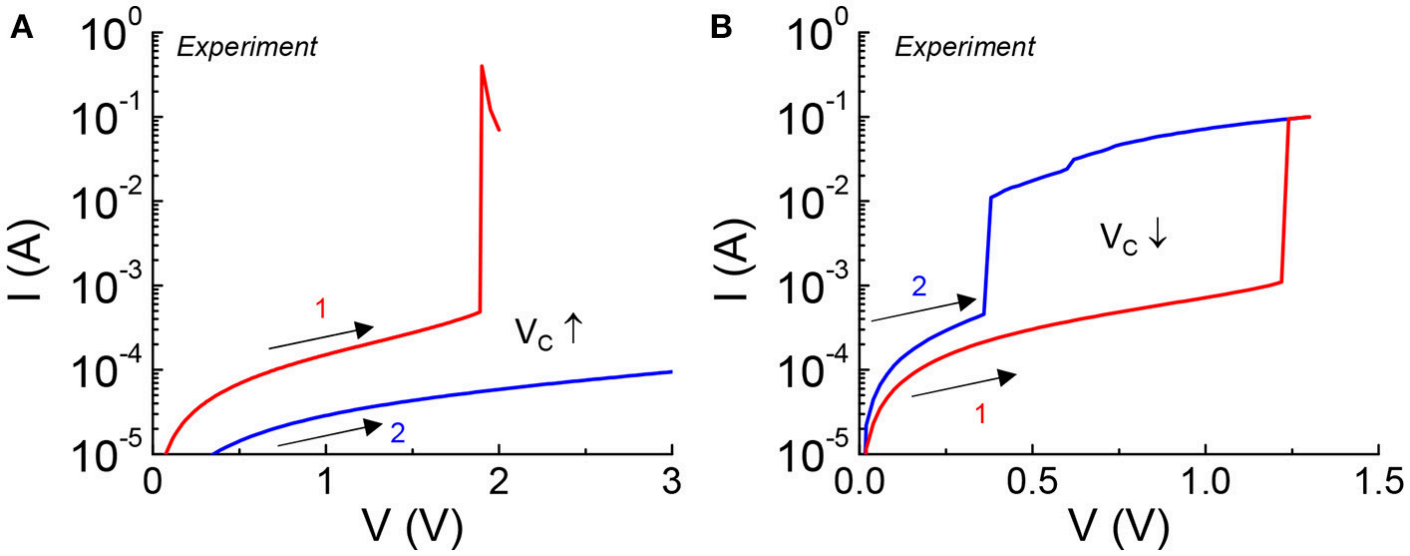
VO_2_ device under excessive DC stress experiencing non-reversible change in critical transition voltage. Two consecutive sweeps are applied to the VO_2_ device with the first sweep stresses the device. **(A)** Increase in V_c_ is caused by an increase of HRS resistance, +ΔR. **(B)** Reduction in V_c_ is the indication of a drop in HRS resistance, –ΔR.

Excessive bias stress to the VO_2_ device can result in non-reversible damage to the material which is manifested in a permeant change in critical transition voltage under DC measurement. This can happen when the device is subjected to a bias outside the safe operating criteria. Two forms of non-reversible damages are shown in Figure 2. Two forward DC sweeps (in the positive direction) are carried out consecutively in one device. Figure 2A shows an increase in V_c_ caused by an increase of the HRS resistance (+ΔR). The current drops over the whole range of applied voltage in the second sweep. Figure 2B shows a reduction in V_c_ which is the indication of a drop in the HRS resistance (–ΔR). The current is higher over the span of applied voltage.

## Results

**Figure 3:**
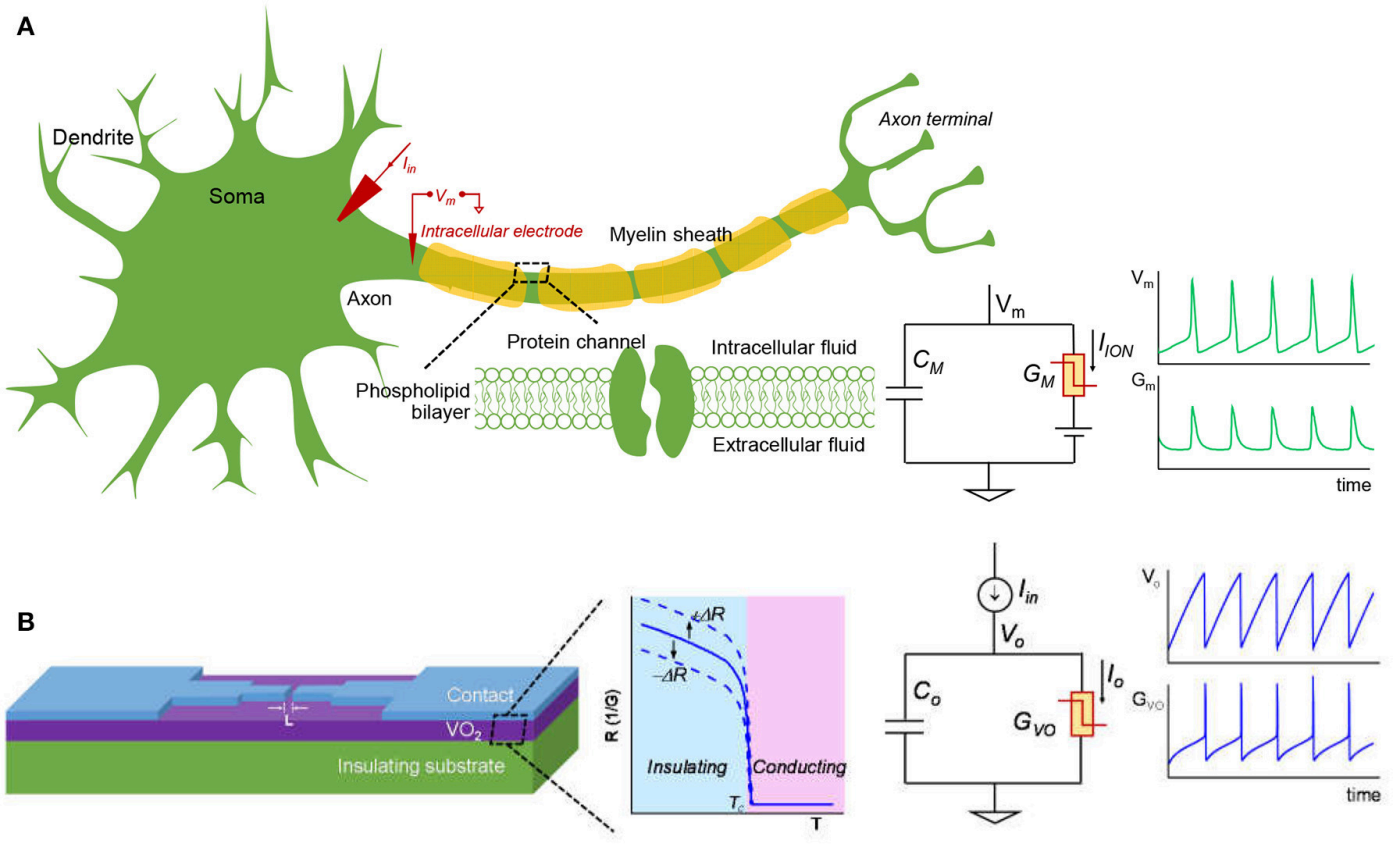
The biological neuron and analogous VO_2_ neuron. **(A)** The membrane of the biological neuron can be viewed as a parallel connection of a membrane capacitance (C_M_) and a membrane conductance (G_M_) that can go through *insulator-to-metal transition* under stimulus. The membrane is polarized at the resting potential due to the different ionic concentration in the intracellular and extracellular fluids. When the neuron is subjected to a steady current clamping, a continuous action potential (AP) is generated, in which the trans-membrane potential (V_M_) and the membrane conductance (G_M_) oscillate. The AP can propagate along the axon and transmit signal to the other connected neurons. The myelin sheath surrounding the axon of some neuron cells can enhance the speed at which impulses propagate. **(B)** A lateral VO_2_ device and a capacitor are used to construct the VO_2_ artificial neuron circuit. The VO_2_ material exhibits a reversible electrothermal *insulator-to-metal transition*. This state change is used to mimic the biological neuron. At constant current input, the VO_2_ neuron output node and VO_2_ conductance oscillate, similar to that of the biological neuron. The insulating-state resistance can be changed when VO_2_ degrades, and this feature is utilized to model spike-timing related neural disorders. Here +ΔR represents an increase of resistance and –ΔR represents a drop in resistance.

Figure 3 shows schematic of a biological neuron and an analogous VO_2_ neuron. The membrane of the biological neuron (Figure 3A) comprises of an insulating phospholipid bilayer that separates the intracellular and extracellular fluids, and protein channels that control the permeation of various ions. As described in the HH model, the neuron membrane is equivalent to a parallel combination of membrane capacitance, C_m_, and transmembrane conductance, G_m_. G_m_ is the sum of various ion channels conductance and it can go through a reversible *insulator-to-metal transition* depending on the voltage across the membrane. An input stimulus can trigger a train of action potentials (AP) that is a temporary reversal of the polarity across the neuron membrane. The AP propagates along the axon through which information is transmitted. In the central nervous system (CNS) such as in the brain and spinal cord, the neuron is myelinated—the myelin sheath surrounds the axon of the neuron cells and promotes rapid signal transmission. Experimentally, the neuron can be stimulated with an input current I_in_. The output AP waveforms depend on the input current, the electrical and geometric parameters of the cell, and the environment such as temperature. The HH model and its parameters are described later in Sec. II.A.

The VO_2_ device and analogous VO_2_ neuron circuit are shown in Figure 3B. The VO_2_ is well-known for its reversible *IMT* proximal to room temperature. Joule heating in two-terminal devices can locally drive the phase change rapidly. This property can be exploited to demonstrate highly non-linear switches (Son et al., 2011; Lin et al., 2017) and artificial neurons (Pickett et al., 2013; Lin et al., 2016; Tuma et al., 2016). The VO_2_ artificial neuron is a circuit that comprises, a minimum of, only two components, the capacitor C_o_ and the conductor (i.e. resistor) G_VO_ as shown in Figure 3B. When the input stimulus I_in_ starts, the VO_2_ neuron exhibits an oscillatory behavior similar to that in the biological neuron. The model and experiment for standalone VO_2_ devices are discussed, respectively in Sec. II.B and Sec. II.C. The VO_2_ neuron circuit is described in Sec. II.D.

### A. hodgkin-huxley (HH) model for biological neuron

**Figure 4:**
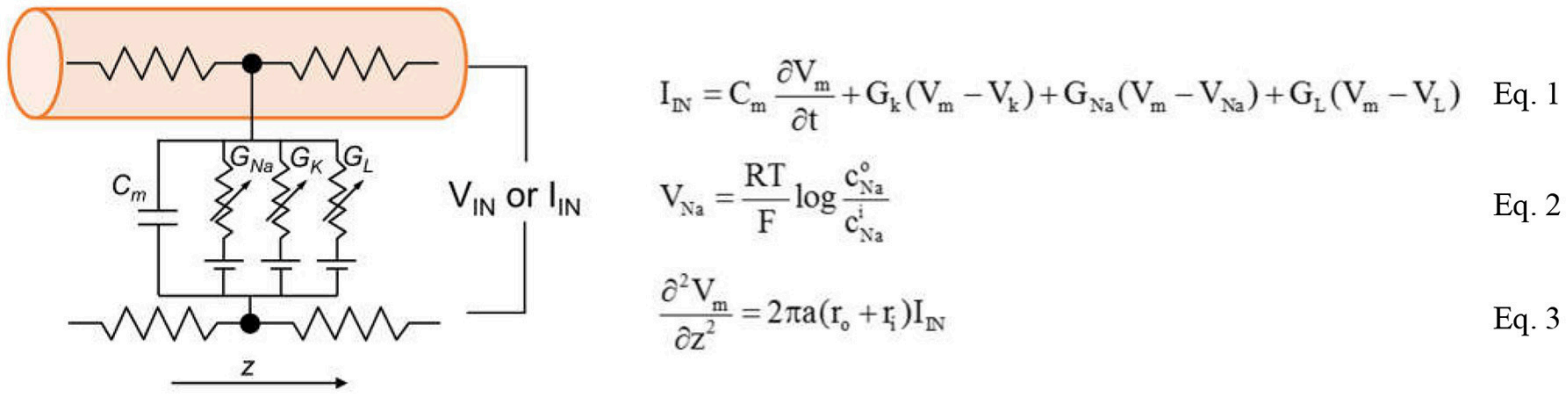
Full schematic of biological neuron that contains two ion channels, leaky capacitive membranes. The equations the form the Hodgkin-Huxley model are shown in the Equations 1–3.

The complete circuit schematic for a patch of the neuron membrane with the HH model is illustrated in Figure 4. The conduction occurs via three channels: The Na^+^ channel, the K^+^ channel and the leakage channel.

The basic mechanisms in the HH model contains ion transport and transmission lines for Action Potential (AP) propagation. The key equations are shown in Equations 1–3. Equation 1 relates the membrane current density I_IN_ to membrane potential V_m_. The area-normalized membrane capacitance is C_m_. Two ion channels with the leaky conductance are included in the model. Their conductance is denoted as G_Na_, G_K_, and G_L_. The Nernst equilibrium potential in Equation 2 relates extracellular and intracellular ion concentrations, respectively denoted as C^i^ and C°. Through Equation 2, the Nernst equilibrium potentials V_Na_ and V_K_ can be obtained for the given Na^+^ and K^+^ concentrations. The molar gas constant R and Faraday's constant F are physical constants. Finally, the propagation of AP along z direction is described by the core conductor equation in Equation 3. It couples the voltage and current along a cylindrical cell where the resistances per unit length inside and outside the cell are, respectively, r_i_ and r_o_. The cylindrical cell has diameter a. The baseline values of the parameters in the HH model are listed in Table 1. The HH neuron model is constructed in Matlab.

**Table 1:** Baseline input parameters for the Hodgkin-Huxley model.

**Parameter**	**Symbol**	**Value**
Conductance of Sodium channel	G_Na_(mS/cm2)	120
Conductance of Potassium channel	G_K_(mS/cm^2^)	36
Leakage conductance	G_L_(mS/cm^2^)	0.3
Extracellular Sodium concentration	C${}_{\text{Na}}^{\text{o}}$(mmol/l)	500
Intracellular Sodium concentration	C${}_{\text{Na}}^{\text{i}}$ (mmol/l)	50
Extracellular Potassium concentration	C${}_{\text{K}}^{\text{o}}$(mmol/l)	20
Intracellular Potassium concentration	C${}_{\text{K}}^{\text{i}}$ (mmol/l)	400
Membrane capacitance	$C_{\text{m}}(\mu {\text{F/cm}}^{\text{2}})$	I

**Figure 5:**
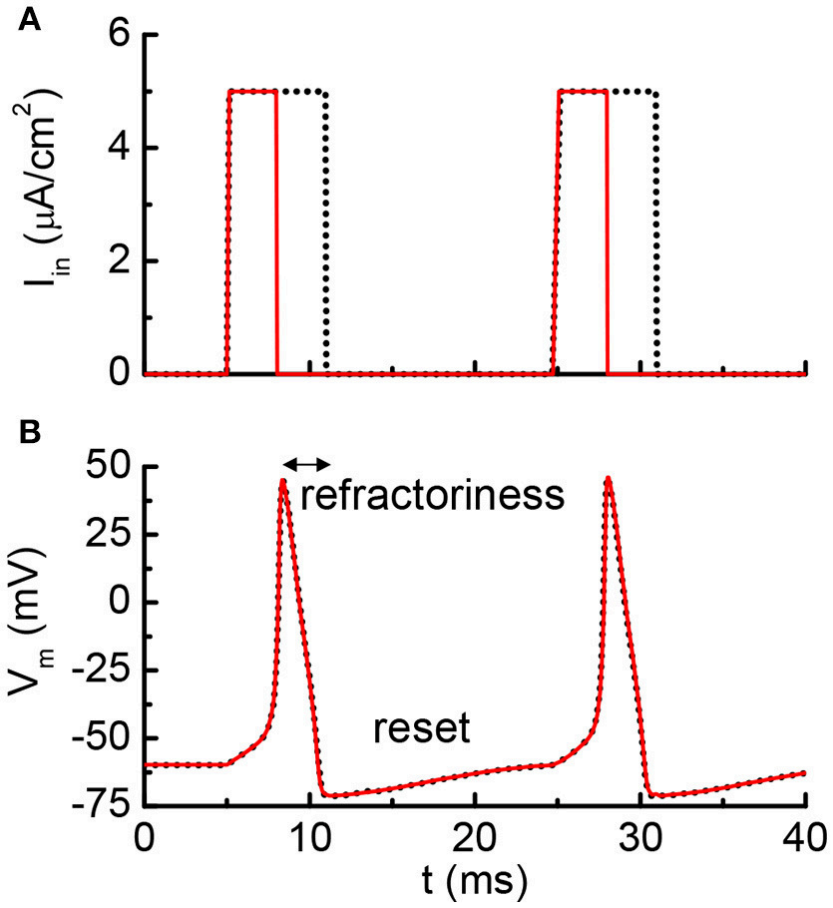
Simulated results from the Hodgkin-Huxley model. **(A)** The neuron is clamped to pulse current input, and **(B)** cross membrane potential. The red solid lines and black dot lines are two different inputs that result in the same firing patterns. The extra current inputs are not being integrated to the membrane capacitance due to the existence of post-refractory period.

Figure 5 shows the results from the HH model with a current clamp. The input is two discrete current pulses. The current-clamped neuron is subjected to the deposition of charge from each of the pulse. The deposited charge of one pulse is merely enough to trigger one neuron firing. The solid red lines are the results for pulse width of 3 ms, and the dashed black lines are for pulse width of 6 ms. The resultant AP profiles are identical for different pulse width, even the long pulse (dashed black) has deposited twice as many charges as the short pulse (solid red). The additional charges are not integrated because it falls into the “refractory period.” A new integration cycle starts only after the neuron resets itself.

### Model for VO_2_ device

**Figure 6:**
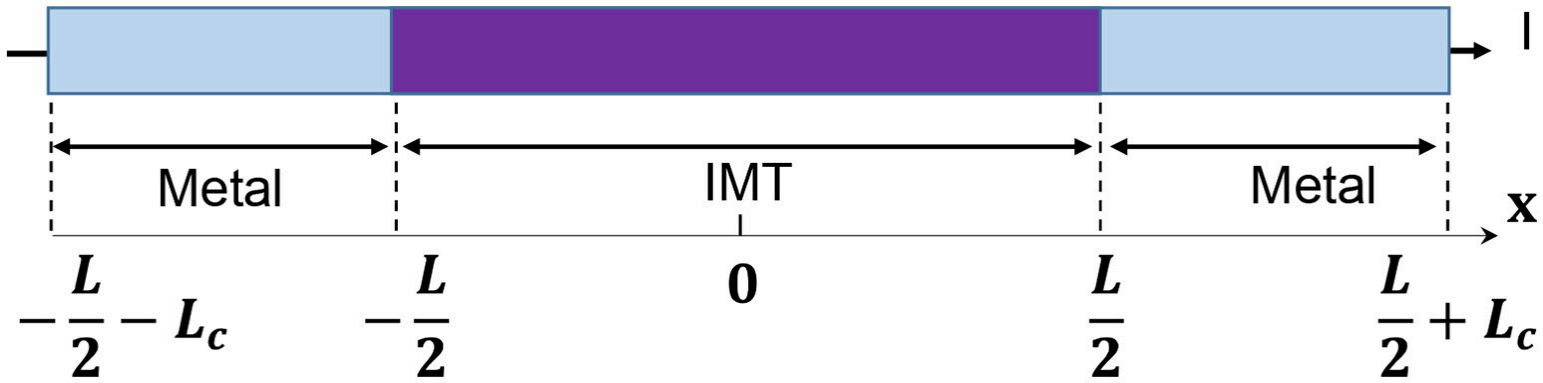
Full schematic of an IMT device with length L between two metal contacts. The device is discretized into segment of dx, etch with its own temperature and resistivity. Heat conduction is along x direction while convective heat loss through the side wall.

The model for electrothermal IMT is first introduced in Lin et al. (2016, 2018), and the relevant detail is discussed in this section. The basic form of heat equation is a parabolic partial differential equation (Equation 4) that describes the relationship of temperature variation in a given volume over time. Equation 4 assumes an isotropic and homogeneous medium in a 3-dimensional space and zero heat flux. The 3D heat transport is simplified by the quasi-1D assumption that the temperature variation perpendicular to the current transport direction along *y* and z is much smaller than that in the transport direction along *x*. This is illustrated in Figure 6 with the current flowing in *x* direction, and the heat equation is converted to Equation 5. Under this assumption, the temperature for a given segment at location *x* and time *t* is obtained as *T(x, t)*. The parameters describing the IMT property are given as follows, specific heat capacity *C*_*th*_, density ρ_*o*_, and thermal conductivity *K*.

(4) $$\begin{array}{rcl} C_{th}\rho_{o}\frac{\partial T}{\partial t} & = & K\left(\frac{\partial^{2}T}{\partial x^{2}}+\frac{\partial^{2}T}{\partial y^{2}}+\frac{\partial^{2}T}{\partial y^{2}}\right) \end{array}$$

(5) $$\begin{array}{rcl} C_{th}\rho_{o}\frac{\partial T}{\partial t} & = & K\frac{\partial^{2}T}{\partial x^{2}} \end{array}$$

There are two origins of heat flux. Firstly, Joule heating results in incoming heat flux to the medium. The power generated by Joule heat follows Ohm's law, and for a unit volume it is:

(6) $$\begin{array}{r} P_{1}=\frac{I^{2}\rho_{r}}{A^{2}} \end{array}$$

where *I* is the total current through the IMT, *A* is the cross sectional area, and ρ_*r*_ is the temperature-dependent resistivity of the IMT. Secondly, the outgoing heat flux is generated by convective heat loss, modeled by the effective convective heat transfer coefficient *h*. Here *h* is assumed to be a constant and is independent of the IMT temperature. Besides *h*, the heat loss through convection for a unit volume is also related to the ambient temperature *T*_*a*_, and IMT's surface to volume ratio *L*_*p*_/A where *L*_*p*_ is the cross sectional perimeter. The power dissipated through side wall heat convection is:

(7) $$\begin{array}{r} p_{2}=\frac{h\cdot L_{p}\left(T-T_{a}\right)}{A} \end{array}$$

Taking into account the heat fluxes, the differential equation for heat transfer is shown in Equation 8.

(8) $$\begin{array}{r} C_{th}\rho_{o}\frac{\partial T}{\partial t}=K\frac{\partial^{2}T}{\partial x^{2}}+\frac{I^{2}\rho_{r}}{A^{2}}-\frac{h\cdot L_{p}\left(T-T_{a}\right)}{A} \end{array}$$

As shown in Figure 6, the IMT with length L is connected to two metal contacts with length *L*_*c*_. As the boundary condition, *L*_*c*_ is assumed to be long enough so that the value of *L*_*c*_ has negligible impact – *L*_*c*_ should be significantly longer than the heat diffusion length. Equation 8 is solved using a numerical method: forward difference for the time domain and central difference for spatial domain. In the spatial domain, the IMT bar is discretized into segment of length *dx*. Each segment has its resistivity ρ_*r*_(*x*). The total IMT resistance *R*_*IMT*_ is obtained by integrating the resistance of all segments:

(9) $$\begin{array}{r} R_{IMT}=\overset{L/2}{\underset{-L/2}{\int }}\frac{\rho_{r}\left(x\right)}{A}dx \end{array}$$

The current through the IMT can then be obtained by

(10) $$\begin{array}{r} I=\frac{V}{R_{IMT}+R_{S}} \end{array}$$

Where *R*_*S*_ is the series resistance. The IMT resistivity as a function of temperature follows a look-up table of resistivity vs. temperature as measured in the experimental VO_2_ device. A typical example of the resistivity vs. temperature is shown in Figure 3B, which is characterized by the insulator-state resistivity ρ_H_, the metal-state resistivity ρ_L_, and the critical transition temperature (T_c_). The baseline values of other physical parameters are listed in Table 2.

**Table 2:** Input parameters for the coupled electrical-thermal model IMT model.

**Parameter**	**Symbol**	**Value**
Thermal conductivity	K (W/K-m)	6
Specific heat	C_th_ (J/K-kg)	690
Effective convective heat transfer coefficient	h (W/K-m^2^)	10
High resistivity state	ρ_rH_ (Ω-m)	10–^3^
Low resistivity state	ρ_rL_ (Ω-m)	10–^5^
Density	ρ_d_ (kg/m^3^)	4 × l0^3^
Cross sec′ area	A (m2)	1 × 10^−12^
Length	L (m)	5 × l0^−7^
Ambient temperature	T_a_(K)	300
Series resistance	R_s_ (Ω)	70

### VO_2_ neuron circuit

**Figure 7:**
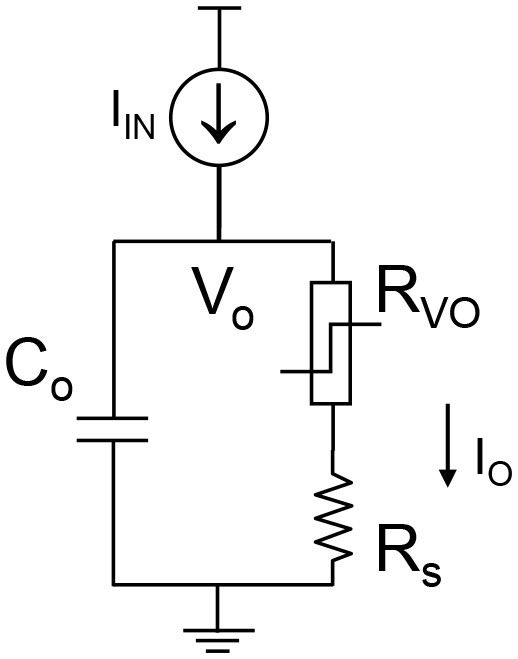
The complete VO_2_ neuron circuit. The whole circuit contains three elements: a capacitor as well as the VO_2_ device with a sensing resistor in series. The output current is sensed by the sensing resistor. The voltage at the capacitor node is denoted as V_o_. It is also the input node for the injected current.

The complete VO_2_ neuron circuit is shown in Figure 7. This is one of the simplest artificial neuron circuits that has been reported, which comprises of only two or three elements. Despite its simplicity, it exhibits striking similarity to the biological neuron (Figure 3A). To investigate the many unexplored functions of the artificial neuron, particularly its connection to neurological diseases, a physical neuron model is imperative.

**Figure 8:**
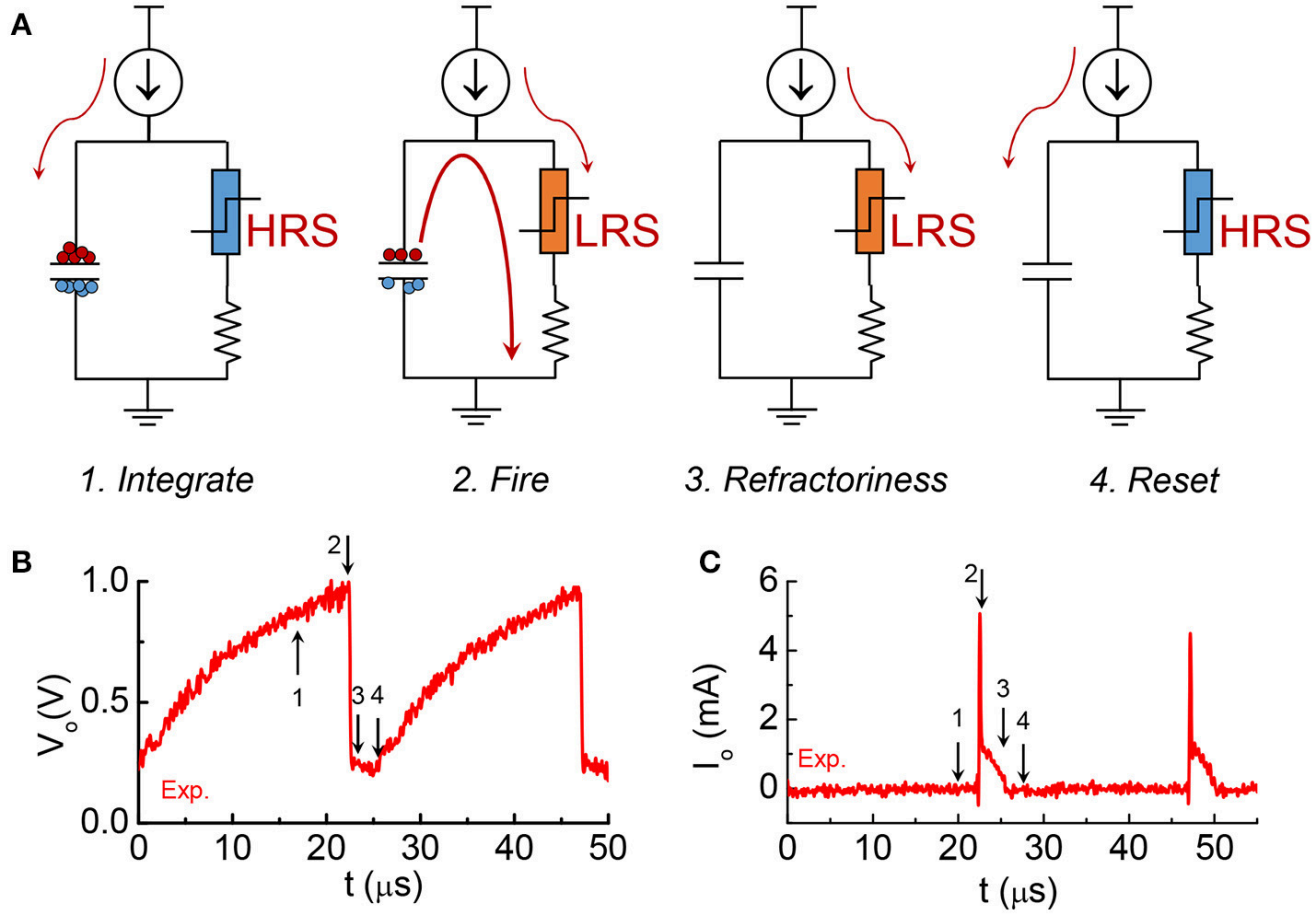
The four stages in one spike cycle in the VO_2_ neuron and the corresponding experimental output waveforms. **(A)** At the integration stage, current is integrated to the capacitance. Very small current goes through the resistor at the right branch as the VO_2_ is at HRS. Voltage at across the capacitor V_o_ is increasing. This is stage 1. When V_o_ reaches V_c_, VO_2_ become metallic and it discharges the capacitor. An instantaneous large current spike appears at the output. V_o_ drops sharply. It is stage 2, fire, which is followed by stage 3, refractoriness (refractory period). In stage 3 the VO_2_ remains in its LRS for some time. Any input current will be drained to ground without integrating to the capacitor. After the refractory period, the neuron resets and is ready for another spike cycle (stage 4). **(B)** The experimental output voltage V_o_ for the 4 stages. **(C)** The experimental output current across the resistance for the 4 stages.

The model is focuses on the material properties of VO_2_ that emulate biological neuron functions. A series resistance R_s_ is added in series with the VO_2_ for two reasons. First, it limits the current when the VO_2_ device transitions to the metallic state, and ensures reliable switching. The safe operating design follows the theoretical guideline derived in Lin et al. (2017). Second, it converts the output current to an output voltage which is measured by the oscilloscope. Each spiking event includes the following four steps: integration, fire, refractoriness and reset as discussed in Figure 8.

**Figure 9:**
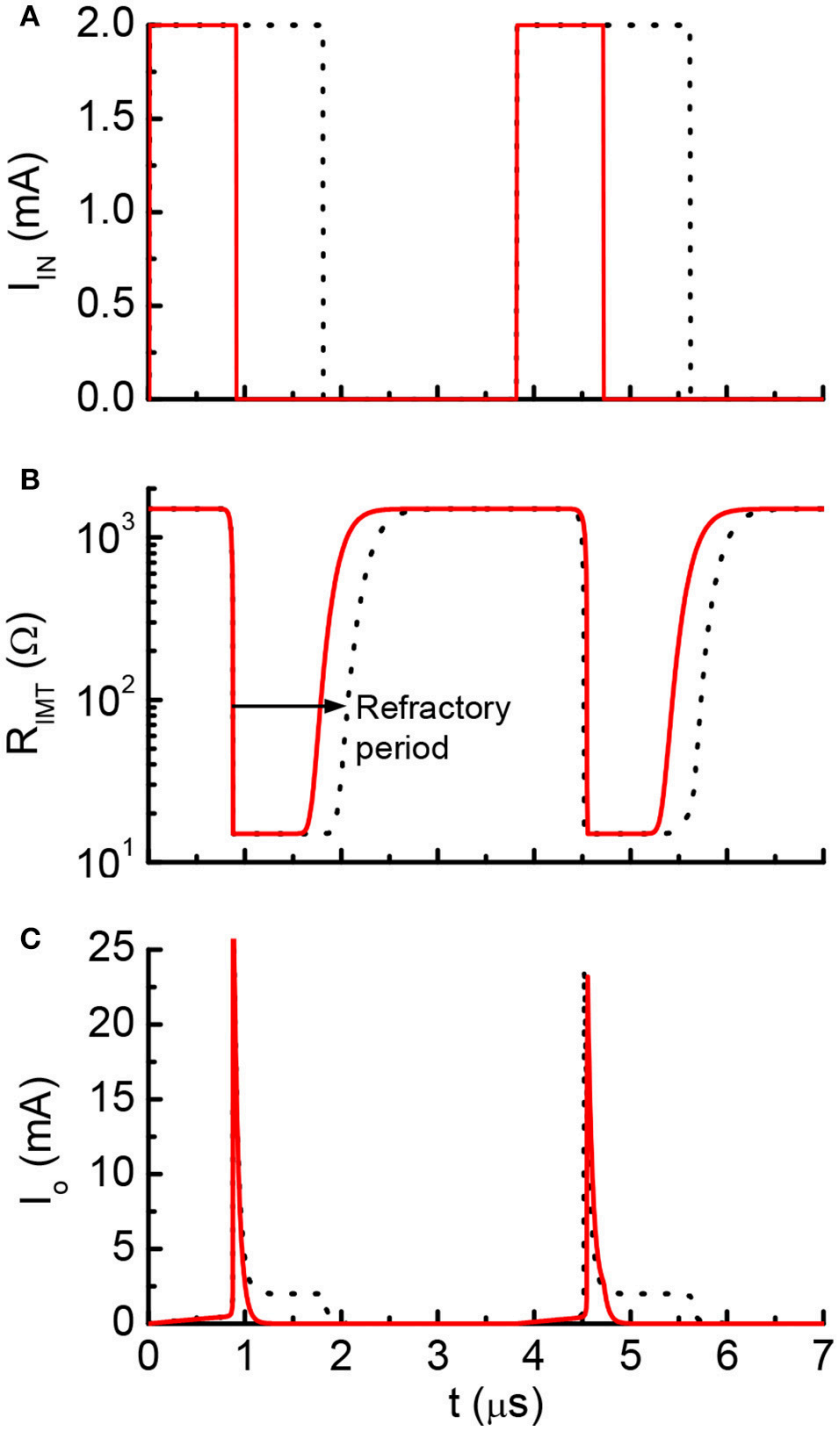
Simulated waveform for current-clamp VO_2_ neuron. **(A)** Input current, **(B)** VO_2_ resistance and **(C)** Output current. The red solid lines and black dot lines are two different inputs that result in the same firing patterns. The extra current inputs are not being integrated to the capacitor due to the existence of post-refractory period. This property directly mimics the biological neuron.

One example of the simulation result is shown in Figure 9. Two discrete current pulses are fed to the input node of the neuron circuits. The deposited charge from each pulse is enough to fire the neuron once. The pulse duration is 0.9 μs for the red solid lines and 1.8 μs for the black lines (Figure 9A). After each neuron firing, the VO_2_ stays in low resistance state for a finite period (Figure 9B). During this period, the input current are directly drained to ground through the VO_2_. The charge is not integrated. As a result, inputs with two pulse durations generates the same firing patterns (Figure 9C). This results show similar post-firing refractoriness as the biological neuron (Figure 5).

Post-firing refractoriness is another important feature in both biological and VO_2_ neurons. A second AP is difficult to be produced immediately following the occurrence of an AP when the cell is regarded to be refractory (Weiss, 1996). Following each firing, the VO_2_ element remains at a temperature above the critical temperature for a time, ~τ_th_+τ_el_, where τ_th_ and τ_el_ are the thermal and electrical time constants, respectively. τ_th_ is related to the thermal mass and heat dissipation. For the electrical time constant τ_el_, it is given by *R*_*met*_*C*_*o*_+*R*_*s*_*C*_*o*_. R_met_ is the metallic-state resistance and R_s_ is the series resistance. Usually, R_met_ is much >R_s_ in normal operation (Lin et al., 2017). To the first order, the refractory period is given by R_s_C_o._ In addition, the continuous high input current can keep the VO_2_ in LRS for longer time. During this period, the VO_2_ element remains in metallic state and new input charge is continuously discharged without being integrated in the capacitor C_o_. Our coupled electrothermal model captures this process and can be used to design the “refractory period” in the VO_2_ neuron circuit. Subsequently, the VO_2_ element resets and starts another integrate-and-fire cycle. The steps mimic the electrically excitable membrane in neuron cells.

## Discussion

### Healthy vs. degenerative neurons, and their VO_2_ analogy

**Figure 10:**
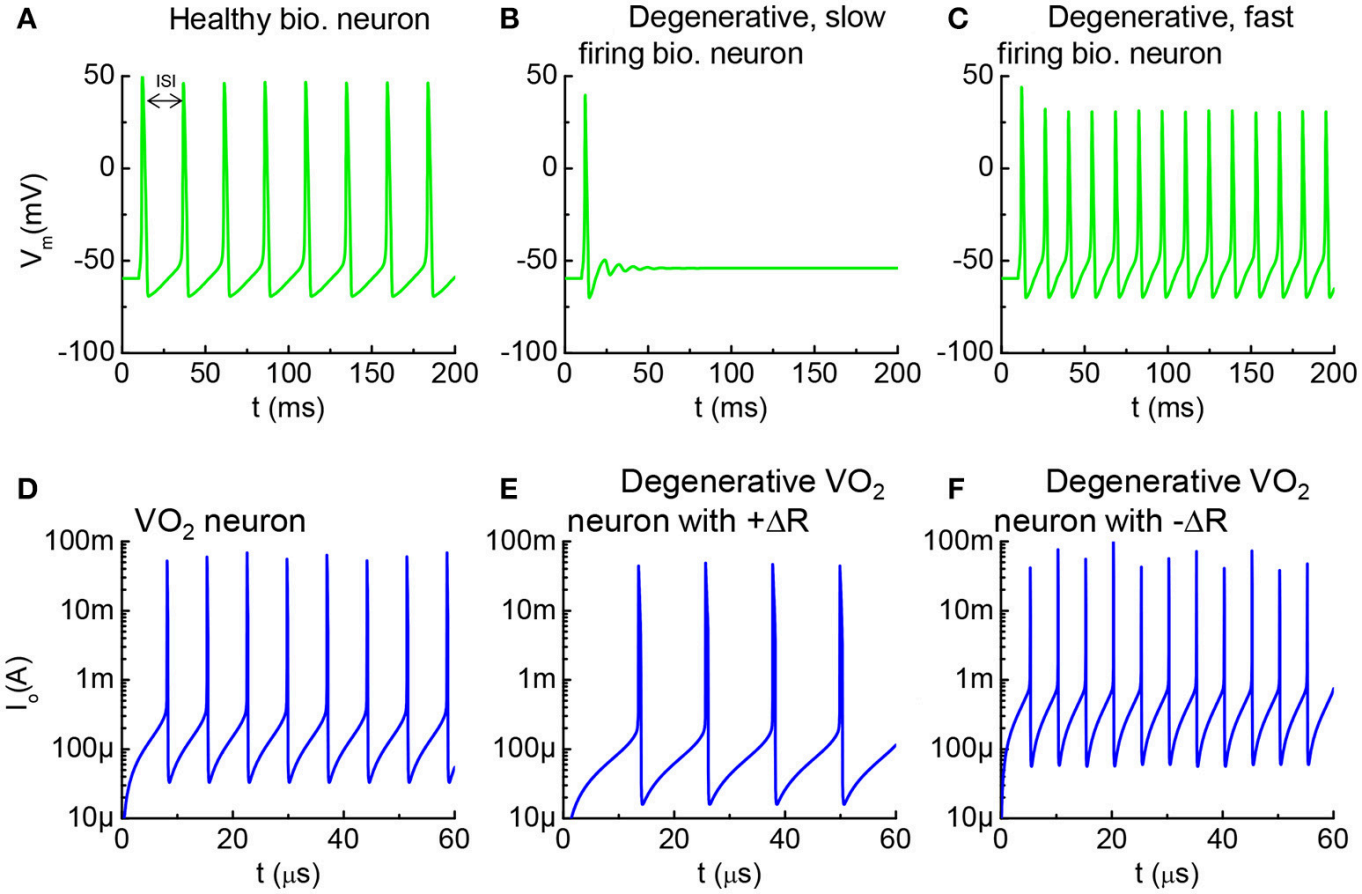
Healthy vs. degenerative fast-firing and slow-firing neurons, and VO_2_ analogy. **(A)** A healthy biological neuron generates AP under a constant current stimulus. The time between two adjacent spikes is termed as the inter-spike interval (ISI). **(B)** A degenerated neuron stimulated by the same input generates AP at longer ISI. **(C)** A degenerated neuron generates AP at shorter ISI. **(D)** Simulation using the VO_2_ neuron model, the intact VO_2_ neuron exhibits oscillatory behavior at a constant input current. **(E)** Simulation of a case where the VO_2_ device is degraded and its insulating state resistance increases (+ΔR). Such degenerative VO_2_ neuron results in longer ISI. **(F)** Simulation of a case where the VO_2_ device is degraded by decreasing its insulating state resistance increases (–ΔR). The leakier VO_2_ neuron results in shorter ISI. **(A–C)** are simulated by the HH model. **(D–F)** are simulated from the VO_2_ neuron model.

In biological neurons, the inter-spiking interval (ISI) is defined as the time interval between two adjacent spikes (Fadool et al., 2011; Okubo et al., 2015). The spiking frequency is the reciprocal of ISI. The AP recorded as a function of time is shown for a healthy neuron (Figure 10A) along with two abnormal neurons (Figures 10B,C), simulated with the HH model. A pathological change of the action potential firing frequency can lead to neurological and psychological disorders. For instance, a decrease in AP frequency is tied to CNS depression and cognitive dysfunction (Friedman et al., 2014). In contrary, an increase in firing frequency is responsible for seizures, pain, ADHD, and anxiety (Wulff et al., 2009). Therapeutic treatment can be designed to restore AP frequency according to the dysfunction mechanisms.

Similar characteristics can be observed in the VO_2_ neurons. The simulation results for three VO_2_ neurons are shown in Figure 10. Figure 10D is the VO_2_ neuron for baseline reference, and Figures 10E,F are the cases where the HRS resistance is modified. ISI for the VO_2_ neuron is defined in the same way as for the case of a biological neuron. The AP frequency reduces if the VO_2_ undergoes a +ΔR degradation, and vice versa. Analytically, the value for ISI can be derived from the VO_2_ neuron parameters as t_ISI_ = C_o_V_c_/I_in_ where V_c_ is the critical voltage to trigger an insulator-to-metal transition in the VO_2_ device under DC I-V measurement and I_in_ is the input current. V_c_ is related to the HRS resistance of the VO_2_ device. Definition of V_c_ is illustrated in Figure 2. Experimentally, we have observed the change of V_c_ (ΔV_c_) due to electrical-stress-induced resistance degradation in the VO_2_ (Figure 7). ΔV_c_ be positive or negative depending on the degradation mechanism. Positive ΔV_c_ indicates an increase in the VO_2_ HRS resistance, and vice versa.

**Figure 11:**
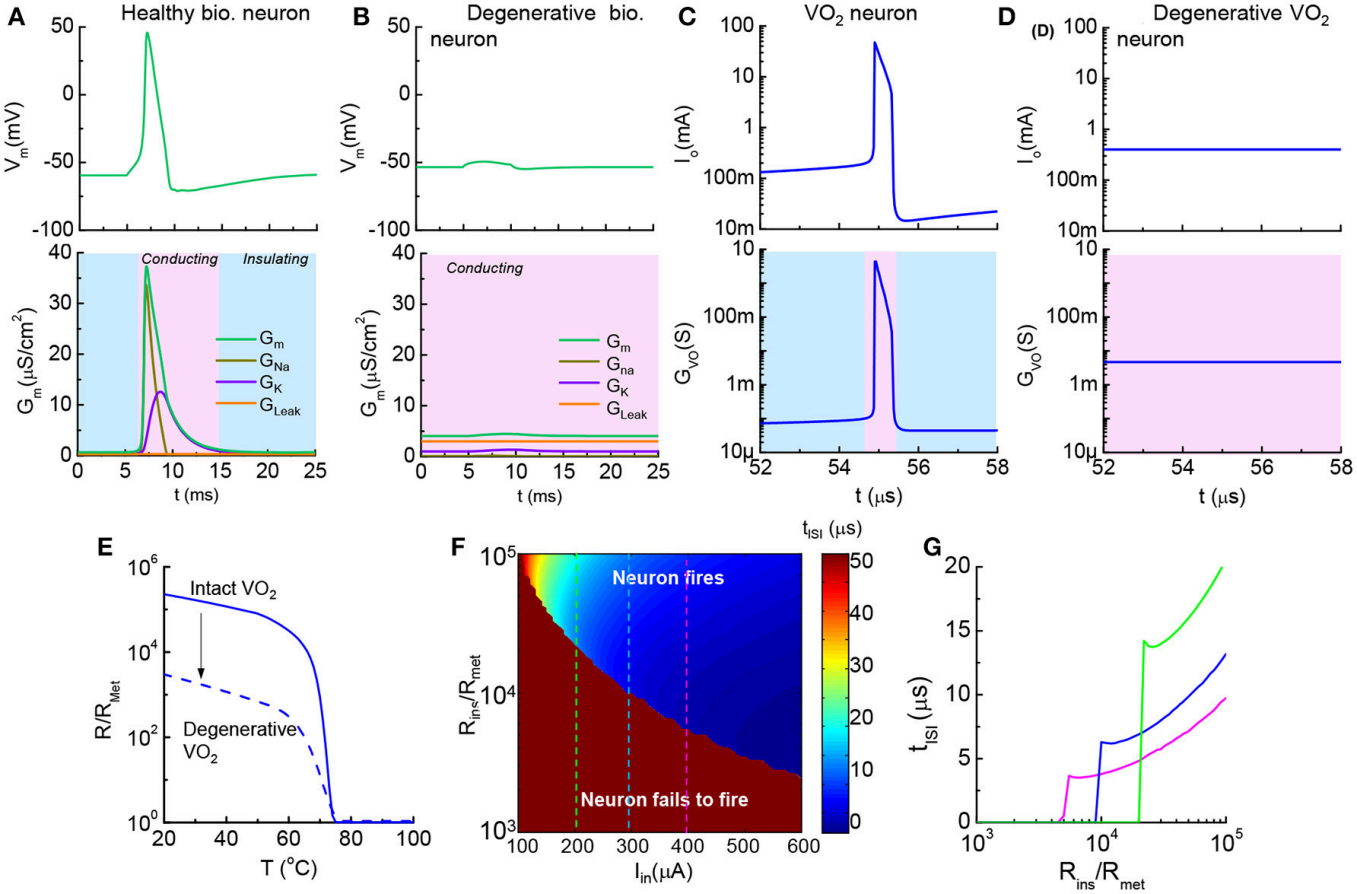
Healthy vs. degenerative leaky neurons, and VO_2_ artificial neurons **(A)**. Healthy biological neuron shows an insulator-to-metal transition during one spike event. G_Na_, G_K_, and G_Leak_ are respectively the Na^+^ conductance, K^+^ conductance and leakage conductance through the membrane, while G_M_ is the the sum of the conductance **(B)**. Degenerative biological neuron with excess leakage G_Leak_, while G_K_ and G_Na_ remain unchanged. No AP spike is observed **(C)**. In one spike of the VO_2_ neuron, the VO_2_ device goes through an insulator-to-metal transition **(D)**. Degenerative VO_2_ neuron with excess leakage (–ΔR) **(E)**. The resistance as a function of temperature normalized to the metallic state resistance (R_ins_/R_met_) illustrates the reduction of resistance of the insulating state by ~100 **(F)**. Contour of ISI shows its dependency on material properties (R_ins_/R_met_) and input stimulus (I_in_). Reduction of insulating-state resistance narrows the neuron operating region for a given input stimulus **(G)**. Three cut lines across I_in_ = 200, 300 and 400 μA in the contour plot **(F)**. **(A,B)** are simulated by the HH model. **(C–G)** are the simulated results from the VO_2_ neuron model.

The pathologically-altered spike timing is linked to other serious degenerative diseases. For example, certain neuromuscular disorders and motor neuron disease (MND) are resulted from the ionic leakage of degenerating membrane and increase of rest conductance (Younger, 1999; Priori et al., 2002). The electrical breakdown of the myelin sheath is one origin for a leaky membrane. Excessive leakage in the membrane makes weak muscles (Wu, 2012). The HH model for biological neuron shows that a neuron fails to fire when conductance is significantly increased (Figures 11A,B). AP generation in a healthy neuron is accompanied by an insulator-to-metal transition in the membrane. However, no transition can be observed in a leaky membrane at the same current input since the charge cannot be integrated effectively.

The increase of conductance in a degenerative, leaky neuron can be modeled in a straightforward manner in the VO_2_ circuit. The resistance vs. temperature of the VO_2_ device is normalized to the low resistance state, R_met_. Figures 11C,D show two neurons at a fixed I_in_: (a), VO_2_ neuron with R_ins_/R_met_ = 10^5^ fires regularly and demonstrates an insulator-to-metal transition (b) while the neuron with R_ins_/R_met_ = 10^3^ fails to fire. The change of VO_2_ properties is shown in Figure 11E.

To provide a systematical perspective on the design of VO_2_ neurons to mimic the corresponding neural disorder, Figure 11F collectively illustrates the impact of HRS resistance and input current on neuron functions. The value of inter-spike interval depends on R_ins_ and I_in_ and is shown as a contour plot. The dark red color is the case where the combination of low input current and small R_ins_ results in failure in spike generation (t_ISI_ → ∞). At a given I_in_, t_ISI_ decreases as R_ins_ drops. When R_ins_ drops to a critical value, the VO_2_ neuron fails to fire (Figure 11G).

**Figure 12:**
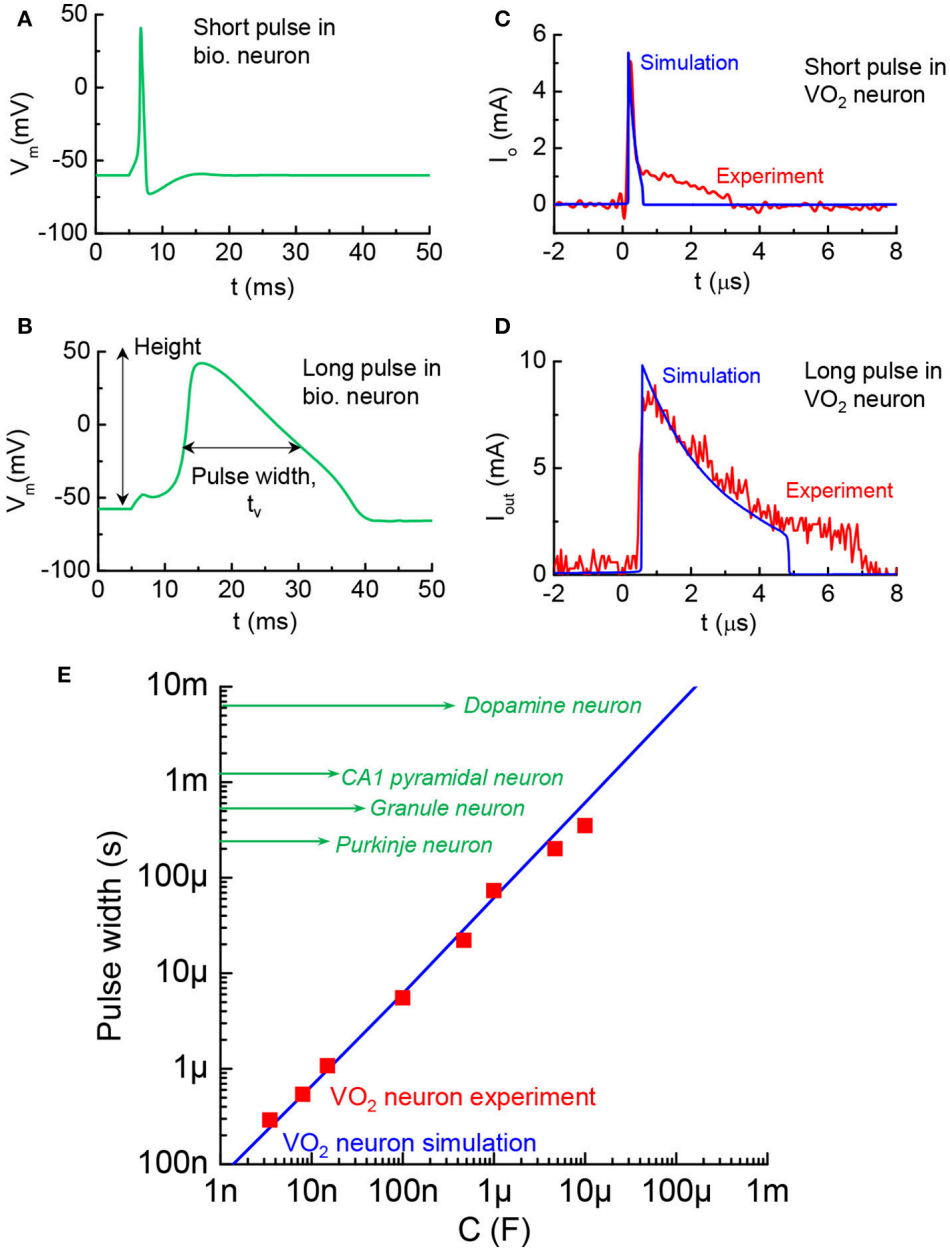
Diversity in AP pulse width across biological neurons, and VO_2_ artificial neuron analogy **(A)**. AP with short pulse width in a biological neuron. The value of t_w_ is taken at full width half maximum **(B)**. AP with long pulse width **(C)**. VO_2_ neuron with short pulse of 0.2 μs. Experiment and simulation show good agreement. The pulse width control is achieved by changing resistance in the circuit **(D)** VO_2_ neuron with long pulse of 2 μs **(E)**. Pulse width of the VO_2_ neuron and the range spans biological neuron studies reported in the neuroscience literature.

The AP pulse width is another distinctive characteristic related to timing in different kinds of mammalian central neurons (Bean, 2007). A short pulse and a long pulse in biological neurons are respectively, illustrated in Figures 12A,B, with the t_w_ change by 10X. The pulse width can be simulated in the VO_2_ neuron by altering the LRS resistance, R_met_, in the VO_2_ devices according to t_w_ = C_o_R_met_. The VO_2_ neuron with short pulses of 0.2 μs and 2 μs are shown in Figures 12C,D. Figure 12E compares the biological neurons and VO_2_ neurons (both simulations and experiments). The AP pulse width can range from a few 100 μs−10 ms which can be matched by the VO_2_ neuron with appropriate capacitance.

**Figure 13:**
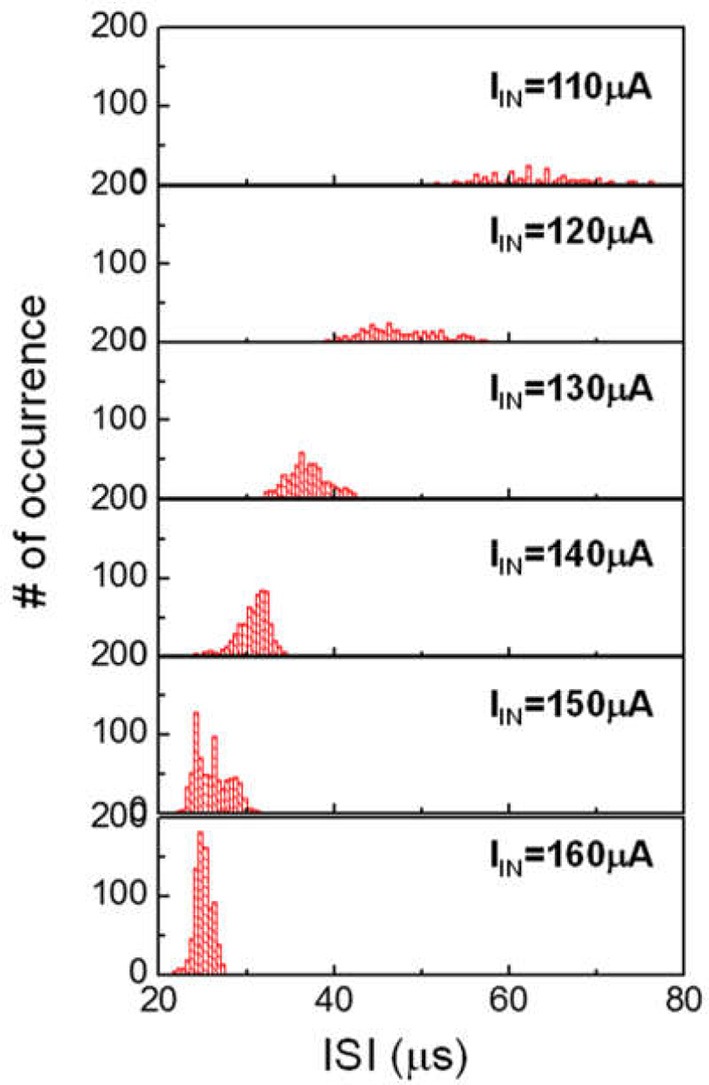
Spike rate (ISI) histogram of VO_2_ neuron for six input currents. In each case, about 500–1000 spike events are measured. The ISI follows the Gaussian distribution. At increased input current, the variance and the mean value of ISI both decreased. These characteristics can mimic the behavior of biological neurons under various current stimulus.

The HH model has been proven to be useful as a fundamental description of neuron behavior (Hodgkin and Huxley, 1952). However, the HH model is known to have limitations. According to HH model, AP spike trains under constant excitation should be uniform. Neurophysiological experiments reveal that the interval of the AP spike (ie ISI) can change. Current clamp experiments show that at low current, the spike rate is random (Fadool et al., 2011). When the injected current is above a certain level the neuron fires continuously at very short and more uniform ISI. The spike rate histogram follows an apparent Gaussian profile. The spike rate histogram for neurons have important implication on the proper function of many animal species. One reprehensive example is the mitral cells in the human olfactory system (Fadool et al., 2011). In a different context, the application of insulin for diabetes treatment and obesity are found to disturb spike rate of the sensory neuron, resulting in incorrect signaling for hunger. Another example is the correlation of neural sequence and learning of vocalization in songbirds. It has been reported that in songbird vocal development, each syllable is produced by a different sequence of action potential bursts in the premotor cortical HVC neurons (Okubo et al., 2015). We can extend our VO_2_ neuron study to emulate such spike rate histogram. Figure 13 shows ISI histogram of VO_2_ neuron for six input currents. The variation of ISI at low input current (I_IN_ = 110 μA) is significant. Such variation reduces as input current increases. The statistical behaviors are related to the cycle-to-cycle operation in VO_2_ device. These characteristics mimic the behavior of biological neurons under various current stimulus.

### Monosynaptic neuron circuit

**Figure 14:**
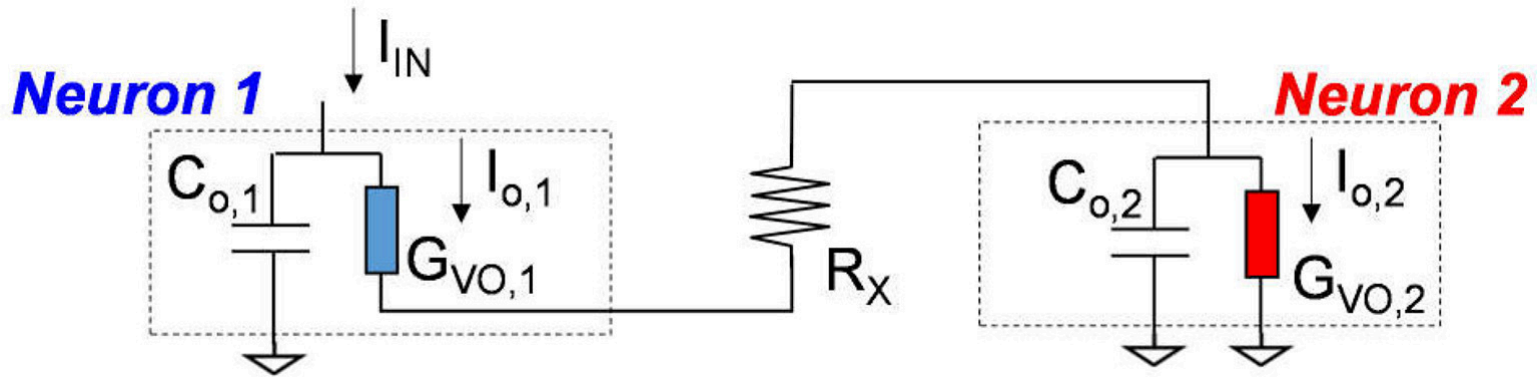
VO_2_ monosynaptic neuron circuit.

We further extend this concept to two-stage cascading neuron circuits in Figure 14. The circuit is a modeling system for the monosynaptic motor neuron in muscular tissue that is responsible for certain motion responses such as the *knee-jerk reaction* that is a model system in neuroscience. Neuron 2 (in red) is the receptive neuron that is driven by Neuron 1 (in blue). Neuron 2 can either fire or not fire depending on the output waveform of Neuron 1 as well as the synaptic resistor R_x_.

**Figure 15:**
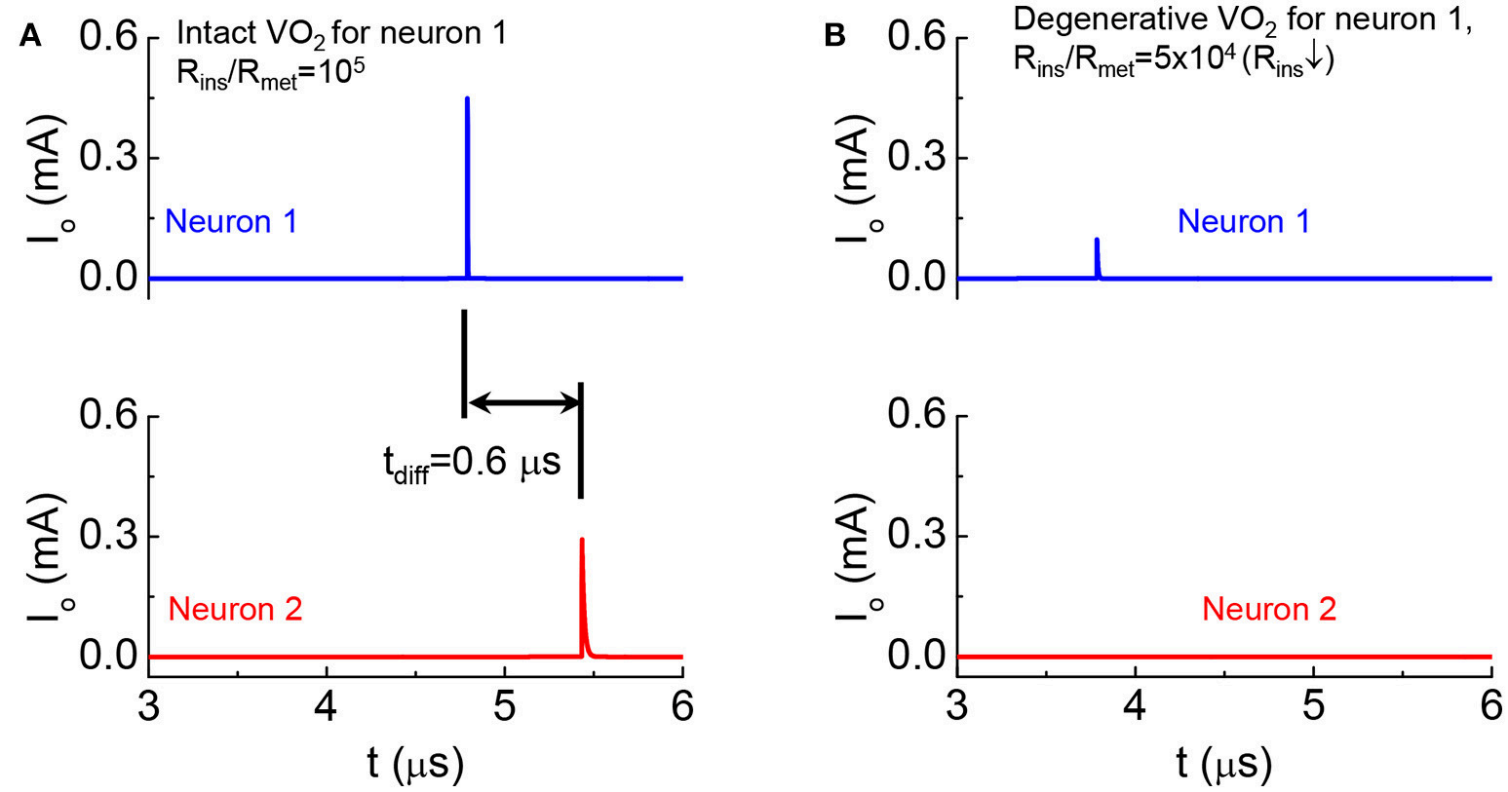
Demonstration of degenerative Neuron 1 leading to the failed signal reception for Neuron 2 in a monosynaptic circuit **(A)**. Output current as a function of time shows the spike events for the intact case **(B)**. Premature spike in Neuron 1 when its HRS resistance is reduced and Neuron 2 fails to spike.

We emulate a monosynaptic circuit that corresponds to the well-known *knee-jerk reaction* used to monitor responses in nerves (Kandel, 2012) as shown in Figure 15. In the two cases for Figures 15A,B, Neuron 2 is the same while the HRS resistance of VO_2_ in Neuron 1 are different. Both neurons are initially at rest. Their temperatures are at equilibrium with the environment and is below the critical transition temperature T_c_. At t = 0, a current is injected to Neuron 1 (see Figure 9). The neuron 1 in case A is intact with high R_ins_/R_met_, The output current as a function of time in Figure 15A shows the spike events for Neurons 1 and 2. The separation of spikes indicate the reaction time (t_diff_ = 0.6 μs) for the signal to propagate between the two VO_2_ neurons. The neuron 1 in case B has a lower HRS resistance. Premature spike in Neuron 1 results in a weak spike and it cannot trigger a spike in Neuron 2 (Figure 15B).

## Conclusion

VO_2_ based circuits can emulate neuronal function and disorders. By carefully varying the electrical properties of the ground state resistance of the artificial neuron, we can precisely identify thresholds for firing and signal propagation that present an analogy to neuronal activity in the brain. While the present study has focused on VO_2_ as a model system, a vast range of threshold switching Mott semiconductors can further be explored in the future.

## Author contributions

JL and SR developed the method, carried out the data analysis, wrote the manuscript. JL and SG fabricated and characterized the VO_2_ devices. JL developed the VO_2_ model and HH neuron model.

### Conflict of interest statement

The authors declare that the research was conducted in the absence of any commercial or financial relationships that could be construed as a potential conflict of interest.
